# Solifenacin versus posterior tibial nerve stimulation for overactive bladder in patients with multiple sclerosis

**DOI:** 10.3389/fnins.2023.1107886

**Published:** 2023-02-21

**Authors:** Nastaran Majdinasab, Neda Orakifar, Leila Kouti, Gholamreza Shamsaei, Maryam Seyedtabib, Mohammad Jafari

**Affiliations:** ^1^Member of Musculoskeletal Rehabilitation Research Center, Ahvaz Jundishapur University of Medical Sciences, Ahvaz, Iran; ^2^Department of Physiotherapy, School of Rehabilitation Sciences, Ahvaz Jundishapur University of Medical Sciences, Ahvaz, Iran; ^3^Musculoskeletal Rehabilitation Research Center, Ahvaz Jundishapur University of Medical Sciences, Ahvaz, Iran; ^4^Faculty of Pharmacy, Clinical Pharmacy Department, Ahvaz Jundishapur University of Medical Sciences, Ahvaz, Iran; ^5^Department of Neurology, Golestan Hospital, Ahvaz Jundishapur University of Medical Sciences, Ahvaz, Iran; ^6^Department of Biostatistics and Epidemiology, School of Public Health, Ahvaz Jundishapur University of Medical Sciences, Ahvaz, Iran; ^7^Department of Neurology, Ahvaz Jundishapur University of Medical Sciences, Ahvaz, Iran

**Keywords:** multiple sclerosis, overactive bladder, quality of life, electrical nerve stimulation, solifenacin

## Abstract

**Introduction:**

Overactive bladder (OAB) is one of the most common complications in patients with multiple sclerosis (MS). Choosing the effective treatment is very important in improving their quality of life (QOL). Therefore, the aim of this study was to compare solifenacin (SS) and posterior tibial nerve stimulation (PTNS) treatment effects in the MS Patients with OAB.

**Materials and methods:**

In total, 70 MS patients suffering from OAB enrolled in this clinical trial study. Patients with a score of at least 3 according to the OAB questionnaire were randomly divided into two groups (35 patients in each group). In one group, patients received SS (5 mg daily for 4 weeks and 10 mg/day for another 8 weeks) and in a second group, patients were treated by PTNS (12 weekly session, 30 min).

**Results:**

The mean (SD) age of patients participating in this study was 39.82 (9.088) and 42.41 (9.175) years for the SS group and the PTNS group, respectively. Patients in both groups showed statistically significant improvements in urinary incontinence, micturition, and daytime frequency (*p* < 0.001). Patients in the SS group had a better response for urinary incontinence after 12 weeks compared to the PTNS group. Also, patients in the SS group reported higher satisfaction and less daytime frequency compared to the PTNS group.

**Conclusion:**

SS and PTNS were effective for improving the OAB symptoms in patients with MS. However, patients demonstrated a better experience with SS in terms of daytime frequency, urinary incontinence, and treatment satisfaction rate.

## Introduction

Multiple sclerosis (MS) is a chronic disease of the nervous system that pathologically characterized by multiple areas of inflammation, demyelination, and glial scarring (sclerosis) in the white matter of the central nervous system (Harbo et al., 2013; Ghasemi et al., 2017). The clinical course of this disease varies from a benign and asymptomatic disease to a rapidly progressive and debilitating disease. MS commonly occurs at young age, and its peak is around 20–30 years of age.

The symptoms of this disease rarely start before the age of 10 years or after 60 years (Goldenberg, 2012; Harbo et al., 2013; Ghasemi et al., 2017). The main characteristic of this disease is the variability of symptoms; it also tends to change in terms of nature and severity over time (4). The common signs and symptoms of MS include muscle weakness, spasticity, reflex changes, sensory disorders, ataxia, nystagmus, vision disorders, autonomic symptoms (e.g., bladder, intestinal, and sexual disorders), and psychological symptoms. The McDonald diagnostic criteria are the best tool for a definitive diagnosis of this disease (Harbo et al., 2013). According to the McDonald diagnostic criteria, the presence of two or more attacks (dissemination in time), along with the clinical evidence and presence of two or more lesions in different parts of the central nervous system (dissemination in place), is necessary for a definite diagnosis of MS. Therefore, the criteria for the diagnosis of MS are based on the patient’s history, clinical examination findings, MRI findings of the brain and spinal cord, and examination of the cerebrospinal fluid.

Bladder and urinary disorders are the common symptoms of MS patients. Bladder care is essential to prevent disabilities, life-threatening infections, and stone formation in these patients. The bladder is probably the only visceral organ with smooth muscles, which is completely controlled by the cerebral cortex. A normal bladder function requires coordination between the sensory and motor components of both autonomic and somatic nervous systems.

Recent advanced studies on neural pathways and neurotransmitters have implicated the presence of neural pathways in the bladder function; therefore, many neurological diseases, including MS, can cause bladder dysfunction (Mahadeva et al., 2014; Khalaf et al., 2015).

The most common bladder dysfunction in MS is excessive bladder contraction. For the treatment of bladder disorders, anticholinergic antimuscarinic agents, such as solifenacin succinate can be used (Ragab et al., 2021). Also, posterior tibial nerve stimulation (PTNS) that involves the stimulation of lower urinary tract nerves often uses to stimulate the nervous pathway of bladder reflex (Staskin et al., 2012). The posterior tibial nerve consists of sensory and motor nerves that directly participates in controlling the sensations and movements of the bladder and pelvic floor muscles (Burks et al., 2010). In PTNS, depolarization of sacral somatic fibers and lumbar afferents indirectly inhibits the activity of the bladder, and can lead to the cessation of bladder contractions (Klingler et al., 2000; Amarenco et al., 2003). Indeed, the PTNS method is based on ancient Chinese acupuncture, which uses acupuncture points on the common peroneal or posterior tibial nerves to inhibit the bladder activity (Geirsson et al., 1993). In spite of available findings, it seems that there is still a need to conduct further research on PTNS. De Gennaro et al. (2011), study highlighted the need for further research on the PTNS. It may be one of the factors which limits the generalizability of previous studies is the selection of samples among patients with a specific disease (Fjorback et al., 2007). Overall, based on previous studies, urinary dysfunctions strongly affect the patient’s mental and psychological health, as well as quality of life (QOL) (Olujide and O’Sullivan, 2005; Mayer and Howard, 2008). According to new reference books, solifenacin succinate is the drug of choice for reducing the symptoms of overactive bladder (OAB). However, due to its side effects and high cost, physiotherapy has been also suggested. Therefore, the present study aimed to investigate and compare the effects of solifenacin succinate and PTNS on the treatment of OAB and improvement of QOL in patients with MS.

## Patients and methods

This clinical trial was conducted on MS patients with OAB in 2019. It was approved by the ethics committee of Ahvaz Jundishapur University of Medical Sciences (code: IR.AJUMS.HGOLESTAN.REC.1399.127) and registered in the Iranian Registry of Clinical Trials (code: IRCT20210207050277N1). Patients with MS, referred to the MS Association of Khuzestan, Iran, were first examined for OAB after confirming the absence of a urinary tract infection *via* urinalysis. This was achieved using the actionable questionnaire, which is an eight-item tool for screening MS patients with OAB. In this method, MS patients with OAB (with scores ≥3) were screened and diagnosed. Finally, according to the inclusion criteria, 70 patients were examined in this study.

The objectives of the study and the benefits of its results were explained to the patients, and they were ensured that they could withdraw from the study at any time. An informed consent form was obtained from all the patients. The volunteers were randomly divided into two groups (35 patients per group). Randomization was carried out by the random block method, where two patients were randomly assigned to each block, and a random code sequence was generated. For the first group, a drug treatment (solifenacin) was applied, while for the second group, PTNS was performed. The solifenacin group consisted of 28 female and 7 male patients, while the PTNS group consisted of 25 female and 10 male patients.

### Inclusion criteria

The inclusion criteria were as follows: (1) a score ≥3 on the actionable questionnaire to confirm neurogenic OAB, causing an emergency need to urinate (which may or may not be accompanied by urge incontinence); (2) absence of a urinary tract infection for at least 1 month before the onset of OAB symptoms; (3) the Expanded Disability Status Scale (EDSS) score of 1–5.5; (4) a minimum interval of 1 month from the last MS attack; and (5) age ≥18 years.

### Exclusion criteria

The exclusion criteria were as follows: (1) pregnancy or planning to conceive in the next 6 months; (2) a history of allergy to solifenacin; (3) cardiac problems (e.g., heart failure); (4) a history of surgery to treat urinary incontinence or other surgeries of the urinary system; (5) stress urinary incontinence or prostatic hyperplasia; (6) diabetes; (7) Parkinson’s disease; (8) a history of anticholinergic drug therapy for OAB in the last month; (9) a history of electrotherapy of the lower limbs and the back; (10) performing the pelvic floor muscle exercise in the last month; and (11) having a heart pacemaker and/or leg prosthesis.

### Data collection

First, the standard actionable questionnaire was completed after confirming the absence of a urinary tract infection *via* urinalysis for screening MS patients with OAB, and MS patients with a minimum score of three on this questionnaire were included in the study.

Subsequently, other questionnaires were completed as scheduled.

#### Actionable questionnaire

This questionnaire is a short eight-item tool, whose validity and reliability have been confirmed. It was designed to screen and identify the problems of MS patients with urinary symptoms (possibly caused by neurogenic bladder) and to accurately identify cases of OAB (score ≥3). This questionnaire contains questions which represent the extent to which patients have experienced the symptoms and what effects they have had on their daily life. The Persian version of this questionnaire has excellent internal consistency (Cronbach’s alpha = 0.91), with an internal correlation coefficient (ICC) of 0.86. The content validity ratio and content validity index of the questions range from 0.66 to 1. Additionally, the following standard questionnaires were completed by the patients at the beginning of the study and 4, 8, and 12 weeks after implementing the intervention.

#### International Consultation on Incontinence Questionnaire in Overactive Bladder (ICIQ-OAB)

This questionnaire was used to determine the severity score of urinary symptoms. Four of the components of this questionnaire were used, that is, the frequency of urination during the day and night and the level of discomfort and the patient’s urgency to go to the toilet and the level of discomfort. The translation and localization of the questionnaire, which has acceptable validity and reliability (0.728), were performed by Sari Motlagh et al. (2015). Each question of this questionnaire has five options, scored from 0 to 4, and the total score is calculated for all questions.

### Multiple Sclerosis Quality of Life Questionnaire-54 (MSQOL-54)

The minimum and maximum scores of QOL in this questionnaire were 0 and 100, respectively, with higher scores indicating higher QOL. The 12 dimensions of this questionnaire are divided into two general dimensions: (1) physical health, including the dimensions of role limitations related to physical problems, physical health, physical pain, vitality, perception of health, and sexual performance; and (2) mental health, including the dimensions of role limitations related to mental problems, subjective vitality, social function, health problems, and life satisfaction. This scale was designed for MS patients by Barbara Vickery at the University of California in 1995. Its validity (content and concurrent validity) has been confirmed in different studies outside (Fjorback et al., 2007; Mayer and Howard, 2008) and inside Iran (Olujide and O’Sullivan, 2005; Tornic and Panicker, 2018). The reliability of this tool was confirmed with a correlation coefficient of 0.86.

The MSQOL-54 contains 54 questions, 18 of which pertain to 14 areas specific to MS (i.e., physical function, role limitations due to physical problems, role limitations due to mental problems, social function, health stress, sexual function, satisfaction with sexual function, pain, energy, perception of health, overall QOL, health changes, cognitive function, and psychological wellbeing); also, 36 questions are related to general QOL. Each question has two to seven options and is rated on a Likert scale. Finally, the score of QOL is determined by combining the scores of the two areas; these two areas include “physical health” and “spiritual-psychological health.” The scores of all 14 areas and two combined areas are in the range of 0–100, with higher scores indicating a better status.

#### International Consultation on Incontinence Questionnaire–Urinary Incontinence Short Form (ICIQ-UI SF)

The validity and reliability of this standard questionnaire were evaluated by Hajebrahimi et al., 2012 in Iran. This questionnaire contains six questions on an individual’s status in the last 4 weeks. Questions 1 and 2 are demographic questions; question 3 pertains to the frequency of urinary incontinence; question 4 measures the amount of urinary leakage; and question 5 measures its impact on QOL. The scores of questions 5, 4, and 2 represent the patient’s real score; question 6 pertains to the time and type of urinary leakage, which was not considered in this study. The total score of the questionnaire ranges from 0 to 21 (mild, 5–1; moderate, 12–6; severe, 18–13; and very severe, 21–19), with higher scores indicating an increase in the intensity of urinary incontinence. The questionnaire was self-administered; if the respondent was illiterate, it was completed with the researcher’s assistance.

### Drug intervention

Oral tablets of solifenacin succinate (5 mg), under the brand name “V-SOL” (Dr. Abidi Pharmaceuticals, Tehran, Iran), were used for drug treatment. This drug, produced in 2004, is one of the antimuscarinic agents, which functions by reducing the bladder contractions. It was initially prescribed at a dose of 5 mg daily for 4 weeks. Its dose was then increased to 10 mg per day (5 mg twice a day) and continued for 12 weeks.

### Posterior tibial nerve stimulation

Electrical stimulation of the posterior tibial nerve was performed using a two-channel TENS 710 device (model 1395, Iran). The characteristics of the current were as follows: intensity, 0.5–10 mA; frequency, 20 Hz; and pulse length, 200 μm. The current intensity increased up to the threshold of motor nerve stimulation, which was determined by the big toe flexion. So far, no studies have reported any side effects for the surface electrical stimulation of the posterior tibial nerve. To perform electrical stimulation, a negative electrode (cathode) was placed behind the inner ankle of the body, and a positive electrode (anode) was placed 10 cm above the cathode. Twelve posterior tibial nerve stimulation sessions (30 min per session) were held over 12 weeks (one session per week).

### Sample size calculation and sampling methods

After entering the study, the patients were randomly divided into two groups. Considering the longitudinal design of the study, the sample size was based on previous studies (Tornic and Panicker, 2018). Considering a type I error of 0.05 and statistical power of 0.95, the correlation coefficient between responses in three measurements was equal to 0.50, the difference in the average response of the two groups was equal to one, and the standard deviation (SD) was equal to one. A sample size of 35 patients per group and a total sample size of 70 patients were finally estimated.

### Statistical methods

To describe the data, mean and SD were measured for quantitative variables, and frequency (percentage) was measured for qualitative variables. The normal distribution of data was evaluated using Kolmogorov–Smirnov test. Moreover, statistical analysis was performed using Chi-square and independent samples *t*-test to examine the distribution of demographic variables in the two groups. Additionally, a generalized estimating equation (GEE) method with an unstructured correlation matrix was used to analyze longitudinal data. Four factors, including group, gender, time, and interaction of time and group, were included in each model. All analyses were performed in SPSS version 25 at a significance level of 0.05.

## Results

The mean age of the patients was 39.82 ± 9.088 years in the solifenacin group and 42.41 ± 9.175 years in the PTNS group; however, there was no significant difference between the two groups (*p* = 0.249). Two patients from the solifenacin group were excluded due to severe constipation. Also, one patient from the PTNS group was excluded due to death because of coronavirus disease. In the solifenacin group, 7 patients (21.2%) were male, and 26 patients (78.8%) were female, while in the PTNS group, 10 patients (29.4%) were male, and 24 patients (70.6%) were female; nevertheless, there was no significant difference regarding the distribution of patients in terms of gender (*p* = 0.441). Urinary incontinence due to solifenacin treatment increased from 2.97 ± 16.33 on the first day to 3.06 ± 5.97 in the 12th week; other findings are presented in Table 1.

**TABLE 1:** The result of generalized equation estimation (GEE) model for trend of changes in each factor over 3 months follow-up.

Dependent variable	Group	Mean ± SD				GEE model	
		Day 1	Week 4	Week 8	Week 12	β	*p*-Value
Urinary incontinence	S	16.33 ± 2.97	12.3 ± 3.44	8.90 ± 3.51	5.97 ± 3.06	-1.406	<0.001*
	P	14.35 ± 3.77	11.41 ± 3.89	9.47 ± 3.70	8.21 ± 3.66		
Physical health	S	53.22 ± 20.04	53.68 ± 19.81	56.21 ± 18.35	59.78 ± 17.60	-0.666	0.163
	P	48.22 ± 15.24	50.08 ± 14.92	52.56 ± 15.07	56.53 ± 15.07		
Mental health	S	45.84 ± 20.65	46.98 ± 19.77	49.06 ± 19.50	52.18 ± 19.75	-0.625	0.344
	P	44.69 ± 18.71	47.03 ± 17.86	49.64 ± 16.68	52.31 ± 16.45		
Micturition frequency during the day	S	2.88 ± 0.89	2.58 ± 0.97	1.88 ± 0.96	1.09 ± 0.91	-0.029	0.612
	P	2.68 ± 1.04	2.41 ± 1.05	1.68 ± 0.98	1.06 ± 1.01		
Suffering from micturition during the day	S	8.85 ± 1.50	6.79 ± 1.84	4.91 ± 1.84	3.42 ± 2.60	-0.884	<0.001*
	P	8.50 ± 1.60	6.24 ± 1.94	6.88 ± 2.09	5.18 ± 2.32		
Micturition frequency during the night	S	3.00 ± 0.87	2.58 ± 0.83	1.67 ± 0.89	1.33 ± 0.89	-0.095	0.338
	P	2.69 ± 0.84	2.41 ± 0.82	1.76 ± 0.99	1.53 ± 0.99		
Suffering from micturition during the night	S	9.03 ± 1.72	7.21 ± 2.22	5.21 ± 2.64	3.91 ± 3.21	-0.106	0.818
	P	7.76 ± 2.19	6.68 ± 2.37	5.97 ± 2.74	4.74 ± 3.22		
Urgency	S	2.91 ± 0.95	2.55 ± 0.97	1.85 ± 0.87	1.36 ± 0.78	-0.082	0.441
	P	2.97 ± 0.90	2.68 ± 0.95	2.03 ± 0.90	1.68 ± 0.94		
Suffering from urgency	S	8.33 ± 2.13	7.88 ± 2.19	4.70 ± 2.88	4.03 ± 3.22	0.315	0.465
	P	8.35 ± 2.10	7.76 ± 2.32	5.68 ± 2.97	4.74 ± 3.08		
Incontinence	S	2.24 ± 0.97	1.91 ± 0.63	1.12 ± 0.78	0.70 ± 0.85	-0.031	0.748
	P	2.41 ± 0.89	2.12 ± 0.69	1.44 ± 0.89	1.03 ± 1.03		
Suffering from incontinence	S	7.97 ± 2.38	6.82 ± 2.27	4.45 ± 3.27	3.06 ± 3.71	-0.241	0.496
	P	8.53 ± 1.21	6.91 ± 2.21	5.38 ± 3.17	4.06 ± 3.89		

S, solifenacin; P, PTNS; SD, standard deviation; β, coefficient of interaction between time and group in GEE model adjusted for group, time, and gender variables; *p-value, significant level at 0.001.

**FIGURE 1:**
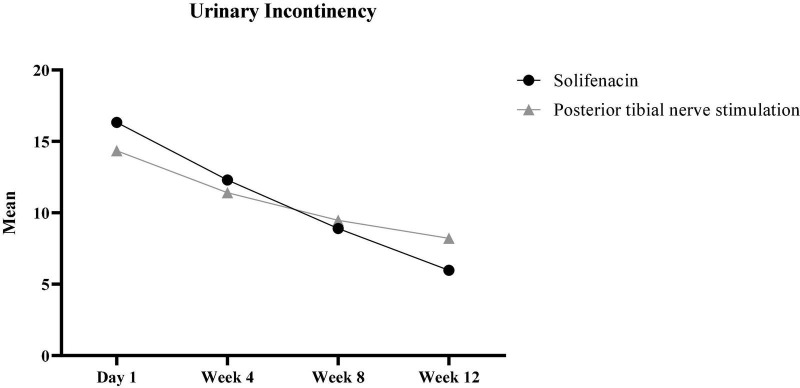
The trend of changes in urinary incontinence during the 3-month follow-up.

Based on the results of GEE models, there was no significant difference between males and females for any dependent variables (*p* > 0.05). The findings showed that the trend of changes during the follow-up was not statistically significant for “physical health” and “Mental health” between the two groups (*p* > 0.05). Also, this result was established for other variables except for “urinary incontinence” and “Suffering from micturition during the day” (Table 1). The results showed that there were significant interactions between time and group for two factors, including “urinary incontinence” (*p* ≤ 0.001) and “suffering from micturition during the day” (*p* ≤ 0.001). In other words, there was a statistically significant difference in the urinary incontinence variable between the two groups over time (Figure 1). Based on the findings, the mean of urinary incontinence in the Solifenacin group was 1.41 units less than another group (β = −1.406, *p* < 0.001).

**FIGURE 2:**
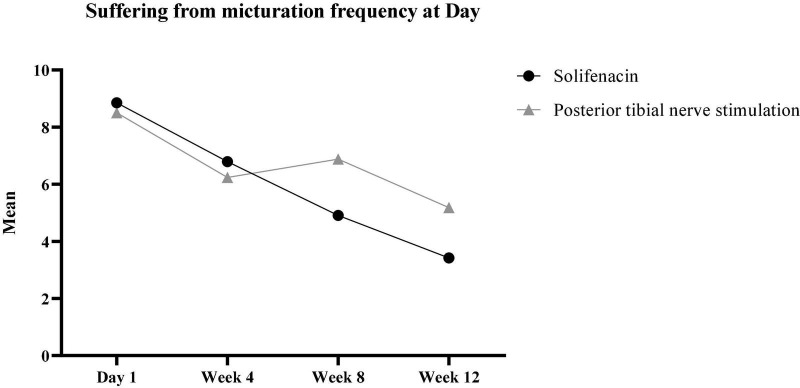
The trend of changes in the micturition frequency during the 3-month follow-up.

Also, the trend of changes in the variable suffering from micturition frequency during the day in the two groups over time was accompanied by a statistically significant difference (β = 0.884, *p* < 0.001). Based on the results, the patients in the Solifenacin group expressed less dissatisfaction with the number of urinations during the day compared to the patients in the PTNS group (Figure 2).

## Discussion

In the present study, the effect of surface electrical stimulation of the posterior tibial nerve and drug therapy (solifenacin) on the treatment of OAB and improvement of the QOL of patients with MS was investigated. To the best of our knowledge, comparison of solifenacin and PTNS therapeutic effects has not been performed on the MS patients in previous studies. The results showed significant between-group differences in the severity of urinary incontinence and the level of inconvenience due to the decrease in frequency of urination at night (for 12 weeks), while there were no significant differences regarding other variables. Also, the findings demonstrated no significant difference in the treatment effects between males and females in this study.

The present results are consistent with some previous studies examining the effects of drug therapy (solifenacin) and PTNS (Al-Danakh et al., 2022). In a study by van Rey and Heesakkers (2011), using 8.8 mg of solifenacin for 8 weeks led to a significant decrease in the number of pads used per day in the MS patients with OAB. Also, the intensity of urinary urgency decreased significantly.

Twenty out of 30 patients chose to continue solifenacin treatment after completing the study. The majority of the patients reported improved QOL. They concluded that solifenacin is effective in the treatment of patients with OAB symptoms (van Rey and Heesakkers, 2011). Besides, Chapple et al. (2006) studied the effects of solifenacin in 2,800 patients with symptoms of OAB, and the findings of this clinical trial showed that a solifenacin dose of 5 mg can be increased to 10 mg once a day. Compared to the placebo, it was effective in reducing incontinence, anxiety, and frequency of urination and increasing the volume of bladder emptying each time; the results of this clinical trial confirmed the use of solifenacin in the treatment of OAB (Chapple et al., 2006).

Additionally, Olujide and O’Sullivan (2005) evaluated the effect of internal stimulation of the posterior tibial nerve by passing a needle through the skin in 37 patients with OAB symptoms and 12 patients with non-obstructive urinary retention problems in 12 weeks. The QOL questionnaire and daily urinary excretion registration forms were used to evaluate the patients. The symptoms improved in 60% of the patients, although QOL improved more significantly in patients with OAB, which is consistent with the results of the present study. The findings of a study by Amarenco et al. (2003) also showed the positive effect of PTNS, which is in line with the current results. In their study, 44 patients with urgent and frequent urinary incontinence and emergencies, caused by an overworked bladder, were selected among patients without any specific neurological diseases, as well as patients with MS, spinal cord injuries, and Parkinson’s disease. Their bladder capacity was compared before and after electrical PTNS. The bladder capacity of 22 patients increased by about 100 ml, which indicates the immediate effect of this intervention; however, in their study, no control group was included to determine the effect of stimulation more precisely, and the participants included both neurological and non-neurological patients. Also, the final results of these two groups were not compared to determine the effect of this intervention more accurately.

Also, Finazzi Agrò et al. (2005) investigated the effect of PTNS over 4 weeks (three sessions every week, 30 min per session) in women with OAB and incontinence. In their study, PTNS was the only intervention used for the treatment group. For the control group, placebo stimulation was performed, and no other intervention with proven effectiveness was used to treat these patients. The results showed that in 71% of the patients in the treatment group, PTNS was an effective intervention. Therefore, similar to the results of the current study, the QOL score increased, and the frequency of urination decreased, whereas in the control group with placebo stimulation, no therapeutic effects were observed.

### Limitations

Although the sample size of this study was sufficient to draw valid conclusions, patients with confounding factors, such as diabetes or Parkinson’s disease, needed to be excluded, and OAB was merely assessed as a complication of MS.

For a better patient response, some physicians may add PTNS to drug therapy. It can be concluded that solifenacin plus PTNS is superior to each intervention used independently (Al-Danakh et al., 2022). Therefore, we only evaluated and compared the efficacy and safety of PTNS versus drug therapy, which is easier to access and less costly.

## Conclusion

The results of the current study showed that both drug therapy with solifenacin and PTNS for OAB reduced the severity of urinary symptoms (e.g., urinary incontinence) and improved the QOL of patients with MS.

## Data availability statement

The original contributions presented in this study are included in this article/supplementary material, further inquiries can be directed to the corresponding author.

## Ethics statement

The studies involving human participants were reviewed and approved by the Ahvaz Jundishapur University of Medical Sciences, Ahvaz, Iran. The patients/participants provided their written informed consent to participate in this study. Written informed consent was obtained from the individual(s) for the publication of any potentially identifiable images or data included in this article.

## Author contributions

NM and NO wrote the manuscript and contributed in conceptualization of the work. LK, GS, and MS collected the samples. MJ provided the ideas and critically edited the entire composition of the manuscript. All authors contributed to the article and approved the submitted version.
